# Relating switching rates between normal and persister cells to substrate and antibiotic concentrations: a mathematical modelling approach supported by experiments

**DOI:** 10.1111/1751-7915.12739

**Published:** 2017-07-21

**Authors:** Gabriel Carvalho, Cyril Guilhen, Damien Balestrino, Christiane Forestier, Jean‐Denis Mathias

**Affiliations:** ^1^ UR LISC Laboratoire d'ingénierie pour les systèmes complexes Irstea Aubière France; ^2^ LMGE UMR6023 CNRS Université Clermont Auvergne Clermont‐Ferrand France

## Abstract

We developed and compared two mathematical models of variable phenotypic switching rates between normal and persister cells that depend on substrate concentration and antibiotic presence. They could be used to simulate the formation of persisters in environments with concentration gradients such as biofilms. Our models are extensions of a previous model of the dynamics of normal and persistent cell populations developed by Balaban *et al*. (2004, *Science* 305: 1622). We calibrated the models’ parameters with experimental killing curves obtained after ciprofloxacin treatment of samples regularly harvested from planktonic batch cultures of *Klebsiella pneumoniae*. Our switching models accurately reproduced the dynamics of normal and persistent populations in planktonic batch cultures and under antibiotic treatment. Results showed that the models are valid for a large range of substrate concentrations and for zero or high doses of antibiotics.

## Introduction

Microbiologists continually grapple with bacterial resistance to antibiotics (Penesyan *et al*., 2015). Antibiotic resistance is an abiding major problem but other mechanisms such as bacterial persistence have gained prominence in recent years and have also emerged as an important factor in the survivability of bacterial populations (Cohen *et al*., 2013). Persister cells (persisters) are stress‐tolerant bacteria in a susceptible isogenic population. They are usually dormant‐like cells able to resume growth quickly on standard media (Balaban *et al*., 2013). Unlike in resistant bacteria, stress tolerance is temporary and reversible: normal susceptible cells switch their phenotype to the persister state and, inversely, persisters switch their phenotype to actively growing susceptible cells. Once persisters switch their phenotype to normal, they lose their tolerance. This phenomenon allows bacterial populations to adopt a bet‐hedging strategy. Consequently, subparts of bacterial populations are able to survive unpredictable stresses and regrow an active susceptible population after stress (Sánchez‐Romero and Casadesús, 2014; Vega and Gore, 2014). The phenotypic switch between normal and persister cells can be caused by the bistability of toxin/antitoxin (TA) modules (Fasani and Savageau, 2013; Gelens *et al*., 2013; Zucca, 2014). Environmental conditions such as starvation, quorum‐sensing, biofilms, subinhibitory antibiotic concentration and diauxic shifts all have the potential to induce persistence (Balaban, 2011; Amato *et al*., 2013; Helaine and Kugelberg, 2014; Harms *et al*., 2016). The activation of stress responses, such as the stringent and SOS responses, has been reported to be involved in tolerant states. Low growth rates have been mathematically modelled to influence the stability of TA modules and increase the formation of persisters (Feng *et al*., 2013). Most studies focus on the HipBA module. However, other TA modules can be involved in bacterial persistence (Kint *et al*., 2012). The number of modules also influences the switching rates between normal cells and persisters (Fasani and Savageau, 2013). The switching from persistent states to normal cells is also affected by the growth medium. Rich media tend to induce the wake‐up of persisters and poor media to inhibit it (Jõers *et al*., 2010).

**Figure 1:**
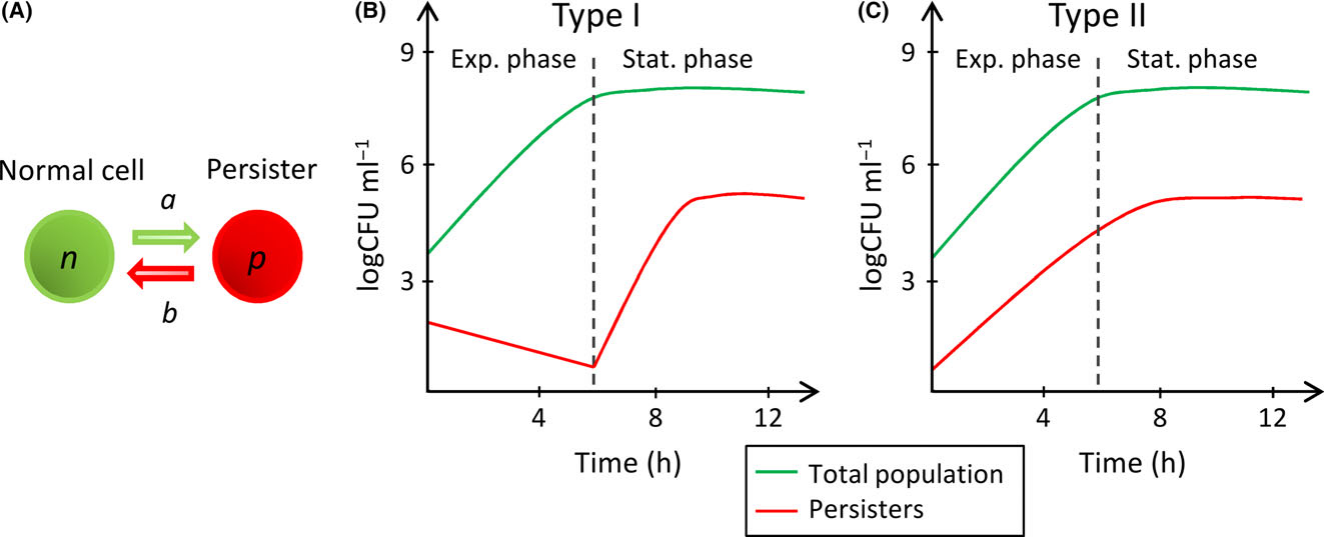
Expected dynamics in planktonic batch cultures. A. A same isogenic bacterial population with two distinct phenotypes forming two subpopulations, normal (*n*) and persister (*p*) cells. *a* and *b* are the switching rates between the two phenotypes. B. Expected dynamics of type I persisters during bacterial growth (Gefen and Balaban, 2009). The switching rate *a* is null during exponential growth (Exp. phase) and increases during stationary phase (Stat. phase) because of substrate limitation (stress). The initial persisters come from the inoculum used to start the batch culture. C. Expected dynamics of type II persisters during bacterial growth (Gefen and Balaban, 2009). The switching rates are constant, and the number of persisters is proportional to the number of normal cells.

Most existing persister models consider two subpopulations, normal (*n*) and persister (*p*) cells (Fig. 1A). Both subpopulations can switch their phenotype from one to another at defined switching rates (Balaban *et al*., 2004; Gefen and Balaban, 2009). For the sake of simplicity, these switching rates are often considered constant (Chambless and Stewart, 2007; Levin and Udekwu, 2010). A few models have related the switching rates to substrate and/or antibiotic concentration(s) (e.g. Roberts and Stewart, 2005; Ayati and Klapper, 2012; Cogan *et al*., 2012; Chihara *et al*., 2015; Szomolay and Cogan, 2014), but were theoretical in nature and not supported by experimental data, or only succinctly (Cogan, 2006). The model of Balaban *et al*. (2004) calculates different sets of switching rates for the exponential and stationary growth phases but does not directly relate the switching rates to environmental parameters. There is a need for an experimentally validated mathematical model that relates switching rates to environmental conditions such as substrate, antibiotic or auto‐inducer concentrations. This kind of mathematical model will help to better understand the dynamics of persisters in heterogeneous environments.

In this work, we tested two mathematical models that relate switching rates to substrate and antibiotic concentrations, two stresses commonly reported in the literature. The models’ parameters were calibrated with experimental killing curves of samples taken regularly from planktonic batch cultures. These experiments assessed the dynamics of normal and persistent populations in batch cultures with variable substrate concentrations and under antibiotic treatment. The validity domain of our models was further assessed by varying the initial substrate concentrations of the experiments and of the simulations. We then compared our models with a reference model with constant switching rates and a model with discontinuous switching parameters between exponential and stationary phases.

## Results

### Dynamics of experimental populations and growth parameters

**Figure 2:**
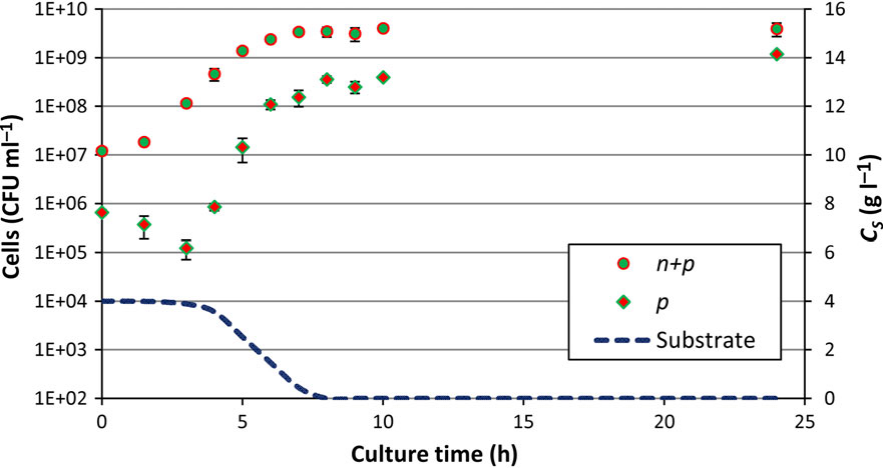
Total (*n* + *p*) and persistent (*p*) populations in the antibiotic‐free batch culture. The persistent population was assessed for each of the three experiments with an initial glucose concentration of 4.0 g l^−1^ separately. The error bars represent the standard deviation of the three experiments. The dynamics of the persistent population shows the pattern of type I persistence. The persister population starts to increase when the substrate concentration (*C*_*S*_) starts to diminish significantly.

The killing curves obtained were biphasic (Fig. S2) and we were able to quantify the persister fraction in the batch culture samples. The dynamics of the normal and persistent populations in the batch culture with 4.0 g l^−1^ of initial glucose are presented in Fig. 2. The initial persisters were formed during the overnight cultures used to inoculate the batch cultures. The evolution of the persistent population occurred in two phases. Between 0 and 3 h of planktonic batch culture, the persister population decreased and was only able to increase after 3 h. This dynamics matches the dynamics of type I persisters predicted in Fig. 1B. However, the persister fraction started increasing before the stationary phase was reached. The results were quite reproducible. The initial decrease in the persister fraction was observed for all individual experiments. The variation between experiments was small compared with the decrease in the persister fraction. With 1.0 and 0.4 g l^−1^ initial glucose, a decrease in the persister fraction was also observed but only between *t*0 h and *t*1.5 h. With these lower initial substrate concentrations, the persister fraction started to increase after *t*1.5 h. With less substrate, persister formation induced by starvation occurred faster.

Table S1 brings together the experimental and optimized growth parameters of the Monod growth model. The *R* ^2^ between the simulated and experimental growth curves was > 0.99 for 4.0 g l^−1^ of initial glucose and was > 0.93 for the other initial glucose concentrations (1.0 and 0.4 g l^−1^). The simulated growth curves were barely affected by the switching model used. An estimation of the substrate concentration in the batch culture is plotted in Fig. 2. The persister population started to increase as soon as the substrate concentration started to decrease significantly, between 3 and 4 h.

### Optimal switching rate parameters

**Figure 3:**
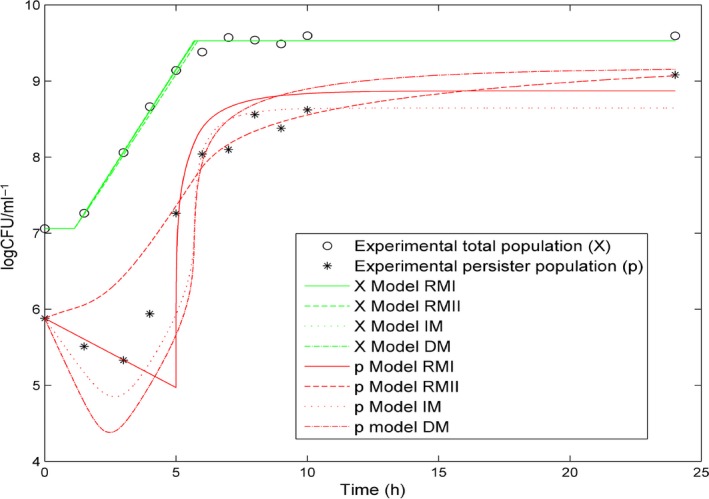
Experimental and simulated total (*X*) and persistent (*p*) populations in an antibiotic‐free batch culture with the different switching models. The initial substrate concentration used is 4.0 g l^−1^. The experimental data plotted is the mean of the three experiments performed with 4.0 g l^−1^ glucose. The switching model selected barely affects the dynamic of the total population. The switching models principally differ by the behaviour of the persistent population in the first five hours of the batch culture.

We optimized the parameters of the four models of switching rates: the discontinuous model of type I persistence (RMI), the model of type II persistence (RMII) and models IM and DM. Parameters were optimized to fit the mean of the three experiments with an initial glucose concentration of 4.0 g l^−1^. The optimal parameters obtained are given in Table 1. The optimal substrate concentration threshold (*C*_*S*,threshold_) of model RMI was 2.4 g l^−1^. For the RMII model, we also tested running optimizations with a growth rate for persisters *μ*_*p*_ **> 0 but the *μ*_*P*_ obtained was negligible and did not affect the other parameters. We left *μ*_*p*_ **= 0, as for the other models. As we assumed *C*_*A*_ **≫* K’* during antibiotic treatments, *K*’ was not assessed. Theoretically, for type I persistence, *a*(*C*_*S*_; *C*_*A*_) is negligible when conditions are favourable. This is consistent with the parameters obtained for the IM and DM models as parameter *a’* is very small. The parameters obtained for the IM and DM models show that substrate limitation is the main trigger that leads to persister formation. *a*_*A*_ is one order of magnitude smaller than *a*_*S*_ for both models. For the IM model, the decay of the persister population during antibiotic treatments was mainly due to the wake‐up of persisters while for the DM model the decay was mainly due to direct killing of persisters by the antibiotic. We cannot tell apart the real mechanism at stake from the dynamics of the populations alone. Figure 3 shows the experimental and simulated dynamics of total and persistent populations in the batch culture with the different models. As anticipated, the choice of the switching model had negligible impact on the total viable cells in the batch culture. Differences were observed in the dynamics of simulated persistent populations. The RMI model showed a sharp change when the substrate threshold was reached whereas the IM and DM models were smoother. The strain used produces type I persisters and the RMII model cannot present the characteristic initial decay of the persistent population, between 0 and 3 h.

**Table 1:** Optimal parameters of the different switching models obtained with 4.0 g l^−1^ glucose

	*a’* (h^−1^)	*a*_*S*_ (h^−1^)	*a*_*A*_ (h^−1^)	*b’* (h^−1^)	*b*_*S*_ (h^−1^)	*b*_*A*_ (h^−1^)	*b*_*SA*_ (h^−1^)	*K* (g.ml^−1^)	*k*_*n*_ (h^−1^)	*k*_*p*_ (h^−1^)
RMI	1.8E‐01			4.2E‐01					1.4E+01	1.9E‐02
RMII	2.2E‐02			2.2E‐14					2.0E+01	5.2E‐01
IM	2.2E‐14	1.2E‐01	1.6E‐02	1.8E‐01	3.6E‐01	6.0E‐01		3.5E‐05	1.3E+01	4.4E‐10
DM	8.8E‐13	7.6E‐02	8.7E‐03	9.8E‐02			1.7E+00	3.5E‐05	1.1E+01	2.2E‐01

**Figure 4:**
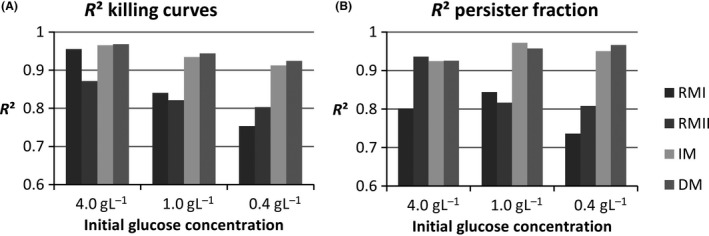
*R* ^2^ obtained for different initial glucose concentrations with the different models. The models’ parameters are optimized for an initial substrate concentration of 4.0 g l^−1^. The models react differently when this initial concentration is reduced. A. *R* ^2^ between the simulated killing curves and the experimental ones. B. *R* ^2^ between the simulated persistent population in the antibiotic‐free batch culture and the experimental one. The IM and DM models are more representative of the experimental data than the RMI and RMII models for the initial glucose concentrations of 1.0 and 0.4 g l^−1^. The DM model gives the best results.

With the optimized parameters in Table 1, we ran two additional simulations by just changing the initial substrate concentration to 1.0 g l^−1^ or 0.4 g l^−1^ and the initial number of normal and persister cells. We compared the results of the simulations with the experimental ones with the same initial glucose concentrations to check the validity of the models in the different conditions. The coefficients of determination (*R* ^2^) obtained for the different models and for the different initial substrate concentrations are given in Fig. 4. Figure 4 groups together the *R* ^2^ between the experimental and simulated killing curves and the *R* ^2^ between the experimental and simulated persistent populations as an additional indicator of the validity of the models. The models were optimized to fit the experimental killing curves. However, they were also able to simulate accurately the persister fraction in the batch culture with the initial glucose concentration of 4.0 g l^−1^. Although the strain used produces type I persisters, the RMII model, used for type II persistence, also produced good results. In Fig. 3, we can observe that the models mainly differ during the first five hours of the batch culture. Although they have different patterns, they all stay close to the majority of the experimental points when the initial substrate concentration is 4.0 g l^−1^. In contrast, all the models do not have the same validity domain. The RMI model was the most affected by substrate concentration changes. When we compared killing curves, the RMII, IM and DM models showed similar losses of accuracy when the initial substrate concentration deviated from 4.0 g l^−1^. When comparing the persister fractions, the IM and DM models appeared to be the least affected by substrate concentration changes. The *R* ^2^ obtained with *C*_*S,i*_ **= 1.0 or 0.4 g l^−1^ were higher than with *C*_*S,i*_ **= 4.0 g l^−1^. Considering the killing curves, the DM model was the best model. Considering the persister fraction, the IM and DM models were just as good.

## Discussion and conclusion

Four models of switching rates between normal and persister cells were tested: RMI, RMII, IM and DM. The IM and DM models were developed to relate substrate and antibiotic concentrations to the switching rates. RMI is a reference model with discontinuous switching rates depending on the growth phase and RMII is a reference model with constant switching rates. The IM and DM models accurately reproduced the experimental data and gave better results than the RMI and RMII models for the different experimental conditions tested. They could be used to simulate persister dynamics in complex environments with substrate concentration gradients.

We fitted a relatively large number of parameters together. As we used numerous initial conditions, we assume that the best local optima obtained for each model are reliable. The optimal parameter sets obtained show that starvation is the main cause of persister formation. The parameter *a’* tends to zero and *a*_*A*_ is an order of magnitude smaller than *a*_*S*_ for both models IM and DM. As reported in previous studies, the stringent response may be involved (Harms *et al*., 2016). As the DM model gave the best results, it is likely that the antibiotic inhibits the wake‐up of persisters despite the presence of substrate. Only experiments at the single cell level would be able to determine the underlying biological mechanism involved, of which there may be several (Helaine and Kugelberg, 2014; Harms *et al*., 2016).

The substrate used in our assays was glucose, and it is likely that changing the substrate will affect the switching rates (Jõers *et al*., 2010; Helaine and Kugelberg, 2014). We also did not take into account oxygen (O_2_) availability and assumed that the use of a shaking incubator did not limit the cells’ access to O_2_. However, O_2_ may play a significant role in complex environments such as biofilms. The stress induced by the depletion of oxygen in deep biofilm layers may increase the switching rate from normal to persisters in addition to the depletion of substrate. Experiments with different oxygen availabilities should be tested, e.g. by lowering the shaking speed for studies in which the hypothesis of non‐limiting oxygen cannot be made. If glucose is indeed the main limiting growth substrate, the IM and DM models give reliable results. With use of the same method, their parameters will need to be calibrated again if other solutes essential for growth are limiting, and the concentration of substrate replaced by the new limiting solute in the equations. The models must be calibrated depending on the environmental conditions of the study conducted.

Another issue is how the switching rates are affected by low antibiotic concentrations. Sub‐MICs have been reported to induce persister formation (Helaine and Kugelberg, 2014) but the parameter *a*_*A*_ obtained was very low for both models IM and DM. In our work, normal cells were rapidly lysed after contact with the high dose of the bactericidal ciprofloxacin antibiotic and did not have the time to switch to persister cells. Additional experiments should be run to determine the parameter *K’* and the validity of the models at low antibiotic concentrations. *K’* is probably a value below the MIC to avoid competition between the death of normal cells at antibiotic concentrations above the MIC and the switch to the persister state. At present, according to our results, the IM and DM models are valid for zero or high antibiotic concentrations.

We considered two subpopulations, normal and persister cells. However, additional persistent subpopulations could be included to explain the dynamics of particular populations, for example, the wild‐type strain in Balaban *et al*., (2004). Persistent populations are quite heterogeneous (Kint *et al*., 2012; Zhang, 2014; Kaldalu *et al*., 2016). Different antibiotics and different combinations of consecutive antibiotic treatments may differently affect a persistent population, suggesting that various kinds of persisters with different tolerance or cross‐tolerance properties can co‐exist in a same culture (Keren *et al*., 2004; Lechner *et al*., 2012). Persisters surviving a given antibiotic may not be the same as those surviving another given antibiotic (or other kinds of stresses). If more than one subpopulation of persisters is observed, each population can have its own model parameters and susceptibility to the antibiotic(s) used. Survival heterogeneity in an isogenic bacterial population can also be due to other mechanisms such as heterogeneity in efflux pump activity among cells (Sánchez‐Romero and Casadesús, 2014), mistranslation of enzyme messengers (Wang *et al*., 2014) or heterogeneity in enzyme production (Wakamoto *et al*., 2013). This heterogeneity between cells may originate from biological noise (Tsimring, 2014). It has also been observed that debris of dead cells can shield viable cells from an antibiotic treatment (Podlesek *et al*., 2016). Although particular mechanisms have been clearly related to persister formation, bacterial persistence is still unclear and may be more widely the reflection of the response diversity of a population (Kahiluoto *et al*., 2014).

In this first study, the IM and DM models accurately reproduced the experimental data. The method developed here could be tested with new strains, limiting substrates or antibiotics. The structure of their equations can be kept and their parameters modified to match new environmental conditions. Knowing the dynamics of persistent populations is crucial to adjusting the timing of antibiotic treatments (Cogan *et al*., 2012). In another context, antibiotics could be used as a means of control to manage ecosystems. Also, the IM and DM models of switching rates can be implemented into models with heterogeneous substrate concentrations, such as biofilm models (see Ayati and Klapper, 2012; Szomolay and Cogan, 2015; Chihara *et al*., 2015).

## Experimental procedures

### Development of a mathematical model with environment‐dependent switching rates

#### Reference mathematical model of the dynamics of persistent populations

Balaban *et al*. (2004) propose that in the case of two subpopulations, normal and persister cells (Fig. 1A), the dynamics of both populations in a planktonic batch culture follow equations (1) and (2). Cells can grow and switch between two phenotypes. *n* is the population of normal cells and *p* is the population of persisters. *μ*_*n*_ and *μ*_*p*_ are the growth rates of the normal and persister subpopulations respectively. *a* is the switching rate towards the persister state and *b* is the switching rate towards the normal state.

(1) $$dn/dt=\mu_{n}n\left(t\right)-an\left(t\right)+bp\left(t\right),$$

(2) $$dp/dt=\mu_{p}p\left(t\right)+an\left(t\right)-bp\left(t\right).$$

Persisters are divided into types I and II (Balaban *et al*., 2004; Gefen and Balaban, 2009). Type I persisters are induced by external stresses. In type I persistence, *a* is assumed to be null in exponential growth. *a* increases with external triggers such as the lack of substrate in stationary phase. In addition, type I persisters have a negligible growth rate, *μ*_*p*_ ≈ 0. In contrast, type II persisters are produced stochastically regardless of the bacterial environment, i.e. *a* and *b* are constant. Type II persisters have been observed to have a positive, though small, growth rate *μ*_*p*_ > 0. The two persister types result in two different kinds of population dynamics during planktonic batch culture (Fig. 1B,C).

The switching and growth rates of equations (1) and (2) (*μ*_*n*_ *, μ*_*p*_ *, a* and *b*) are obtained by fitting the equations to the dynamics of normal and persistent populations in planktonic batch cultures and regrowth experiments (Balaban *et al*., 2004; Lechner *et al*., 2012). The persister fraction in a sample is measured by treating it with antibiotics and counting viable cells over time to obtain a killing curve. A regrowth experiment is the measurement of the regrowth of the survivors of an antibiotic treatment (mostly persisters) over time in a fresh, antibiotic‐free medium. *a* and *b* are considered constant when fitting these equations. This does not matter in the case of type II persistence, as switching rates are always constant (equations (5) and (6)). However, for type I persistence, no continuous model can be used for all growth phases. Different switching rates must be calculated for different environmental conditions. Assuming *a *=* *0 in exponential growth, *b* can be estimated during this time lapse. Then, assuming that *b* remains constant, *a* can be estimated during stationary phase. This results in a discontinuous model for a whole batch culture with different parameters for exponential and stationary phases. If we consider a particular substrate concentration (*C*_*S,threshold*_) to be the threshold between the exponential and stationary phases, we write the switching rates of type I persistence in a batch culture as equations (3) and (4). *C*_*S*_ is the substrate concentration and *a’* and *b’* are constants. We take the following switching models, RMI and RMII, as reference models.

- RMI model: discontinuous model of type I persistence
  (3) $$\text{if}\;C_{S}\geq C_{\left(S,\mathrm{threshold}\right)}\;a=0\;\text{and}\;b=b^{\prime },$$
  (4) $$\text{if}\;C_{S}<C_{\left(S,\mathrm{threshold}\right)}\;a=a^{\prime }\;\text{and}\;b=b^{\prime }.$$
- RMII model: type II persistence
  (5) $$a=a^{\prime }$$
  (6) $$b=b^{\prime }$$

The RMI model is suitable for batch cultures but could be unsuited for environments with concentration gradients such as biofilms. We must extend the model of Balaban *et al*. (2004) to account for environmental conditions and use it for complex environments, where exponential and stationary phases cannot be clearly separated and switching rates are not spatially homogeneous.

#### Extending the current model to include substrate and antibiotic concentrations

As mentioned in the introduction, various environmental conditions can affect switching rates. Substrate limitation and stressful conditions, such as the presence of antibiotics, are common (Balaban, 2011; Helaine and Kugelberg, 2014). We chose to take into account substrate and antibiotic concentrations (*C*_*S*_ and *C*_*A*_) and to relate them to the switching rates *a* and *b*. We assumed the growth rate of persisters to be zero (*μ*_*p*_ = 0) as in the case of type I persistence. As we assume *μ*_*p*_ **= 0, the growth rate of the total population (*μ*) is the growth rate of normal cells, *μ = μ*_*n*_. We adapted equations (1) and (2) by replacing the constant parameters *μ, a* and *b* with the functions *μ*(*C*_*S*_), *a*(*C*_*S*_; *C*_*A*_) and *b*(*C*_*S*_; *C*_*A*_). In addition, in the presence of antibiotic, normal cells and persisters are killed. We added killing rates to equations (1) and (2) to take this factor into account. The new equations that we used to simulate the dynamics of normal and persistent populations in batch cultures were equations (7) and (8). *k*_*n*_(*C*_*A*_) and *k*_*p*_(*C*_*A*_) were the killing rates of normal and persister cells respectively. They are considered constant during antibiotic treatments and set to zero during antibiotic‐free batch cultures.

(7) $$\begin{array}{cc} dn/dt & \;=\;\mu \left(C_{S}\right)n\left(t\right)-a\left(C_{S};C_{A}\right)n\left(t\right)\\ & \;+b\left(C_{S};C_{A}\right)p\left(t\right)-k_{n}\left(C_{A}\right)\;n\left(t\right) \end{array}$$

(8) $$dp/dt=a\left(C_{S};C_{A}\right)n\left(t\right)-b\left(C_{S};C_{A}\right)p\left(t\right)-k_{p}\left(C_{A}\right)\;p\left(t\right)$$

For the switching rate *a*(*C*_*S*_; *C*_*A*_), the literature is unanimous on the induction of the persister state. Starvation or sub‐MIC antibiotic concentrations can separately induce the persister state (Balaban, 2011; Helaine and Kugelberg, 2014; Harms *et al*., 2016). Thus, we assume that *a*(*C*_*S*_; *C*_*A*_) must increase when the substrate concentration decreases or the antibiotic concentration increases. For the switching rate *b*(*C*_*S*_; *C*_*A*_), the literature is unclear whether the substrate and the antibiotic influence the wake‐up of persisters independently or interdependently. In a few models previously described, the switching rate *b* was assumed to be zero in the presence of antibiotic regardless of the substrate concentration (Cogan *et al*., 2012; Szomolay and Cogan, 2015). If *b*(*C*_*S*_; *C*_*A*_) was inhibited by the presence of antibiotic, it would prevent persisters from waking up and dying during an antibiotic treatment. In this case, the substrate and the antibiotic influence *b* interdependently. However, a few experiments tend to prove that the substrate improves the efficacy of antibiotic treatments (Wood, 2016). The substrate could increase the switching rate *b* despite the presence of antibiotic. In this case, the substrate and the antibiotic influence *b* independently.

Thus, we developed two mathematical models to relate the switching rates to the substrate and antibiotic concentrations, *C*_*S*_ and *C*_*A*_ respectively. We developed a model in which substrate and antibiotic concentrations affect *b*(*C*_*S*_; *C*_*A*_) independently (model IM), and one in which substrate and antibiotic concentrations affect *b*(*C*_*S*_; *C*_*A*_) interdependently (model DM). We used Hill functions (such as Monod and Michaelis–Menten equations), which are common in biology, to build the equations of the two models. Switching rates are bounded by a minimum and a maximum, depending on environmental conditions, as observed in previous models (Cogan, 2006; Cogan *et al*., 2012).

- Model IM: Substrate and antibiotic concentrations influence *b*(*C*_*S*_; *C*_*A*_) independently.
  (9) $$\begin{array}{cc} a(C_{S};C_{A}) & \;=\;a^{\prime }+a_{S}\times \left(1-C_{S}/\left(C_{S}+K\right)\right)\\ & +a_{A}\times C_{A}/\left(C_{A}+K^{\prime }\right) \end{array}$$
  (10) $$\begin{array}{cc} b(C_{S};C_{A}) & \;=\;b^{\prime }+b_{S}\times C_{S}/\left(C_{S}+K\right)\\ & +b_{A}\times \left(1-C_{A}/\left(C_{A}+K^{\prime }\right)\right) \end{array}$$
- Model DM: Substrate and antibiotic concentrations influence *b*(*C*_*S*_; *C*_*A*_) interdependently.
  (11) $$\begin{array}{cc} a(C_{S};C_{A}) & \;=\;a^{\prime }+a_{S}\times \left(1-C_{S}/\left(C_{S}+K\right)\right)\\ & +a_{A}\times C_{A}/\left(C_{A}+K^{\prime }\right) \end{array}$$
  (12) $$\begin{array}{cc} b(C_{S};C_{A}) & \;=\;b^{\prime }+b_{SA}\times C_{S}/\left(C_{S}+K\right)\\ & \times (1-C_{A}/\left(C_{A}+K^{\prime }\right)) \end{array}$$

*a’*,* a*_*S*_, *a*_*A*_, *b’*,* b*_*S*_, *b*_*A*_ *, b*_*SA*_ *, K* and *K’* are constants. Equations with the form *Concentration/(Concentration+Constant)* are bounded by 0 and 1. Thus, *a*(*C*_*S*_; *C*_*A*_) is bounded by *a’* and (*a’ *+ *a*_*S*_ + *a*_*A*_) and *b*(*C*_*S*_; *C*_*A*_) is bounded by *b’* and (*b’* + *b*_*S*_ + *b*_*A*_) or (*b’* + *b*_*SA*_). The IM and DM models are consistent with observations made in the literature (Balaban, 2011; Helaine and Kugelberg, 2014; Harms *et al*., 2016): *a*(*C*_*S*_; *C*_*A*_) will increase owing to nutrient starvation or the presence of antibiotics, eventually at subinhibitory concentrations. At stationary phase, substrate limitation will lead to an increase in the persister/normal cell ratio. We could not determine whether the substrate and the antibiotic influence *b*(*C*_*S*_; *C*_*A*_) independently or interdependently. In the IM model, *b*(*C*_*S*_; *C*_*A*_) needs the presence of substrate *or* the absence of antibiotic to increase. In the DM model, *b*(*C*_*S*_; *C*_*A*_) needs the presence of substrate *and* the absence of antibiotic to increase.

To calculate *μ*(*C*_*S*_), we chose a classical Monod growth model. The growth rate *μ*(*C*_*S*_) depends on the substrate concentration *C*_*S*_, the maximal growth rate *μ*_max_ and the Monod constant *K*_*S*_ (equation (13)). The Monod growth model usually assumes *μ*(*C*_*S*_)* *= 0 during the lag phase. The growth rate during the lag phase was determined by equation (14), with *t*_*lag*_ being the duration of the lag phase. With *Y*_*XS*_, the average mass of substrate consumed to produce one cell, substrate dynamics in the batch culture was determined by equation (15). We assumed the consumption of substrate by persisters to be zero. Substrate concentration over time could be related to growth and switching rates: *μ*(*C*_*S*_), *a*(*C*_*S*_; *C*_*A*_) and *b*(*C*_*S*_; *C*_*A*_).

- Growth rate during exponential and stationary phases
  (13) $$\mu \left(C_{S}\right)=\mu_{\max}C_{S}/\left(C_{S}+K_{S}\right)$$
- Growth rate during the lag phase
  (14) $$\text{If}t<t_{\mathrm{lag}}\;\;\mu \left(C_{S}\right)=0$$
- Substrate consumption
  (15) $$dC_{S}/dt=-\mu \left(C_{S}\right)n\left(t\right)\times Y_{XS}$$

With equations (7), (8) and (15), we are now able to simulate the dynamics of normal and persistent populations in a batch culture and under antibiotic treatment. *a*(*C*_*S*_; *C*_*A*_) and *b*(*C*_*S*_; *C*_*A*_) must be replaced by the switching model selected. To calibrate our models’ parameters, we need experimental dynamics of normal and persistent populations in environments with variable substrate concentrations and under antibiotic treatment. A batch culture experiences a diminishing substrate concentration owing to its consumption by growing cells. Switching rates should vary accordingly. To experimentally reveal the persistent population, we must challenge a culture with antibiotic and count the survivors over time. The persister fraction can be quantified by the characteristic biphasic killing curve obtained. Switching rates are also affected by the antibiotic treatments, as assumed by the IM and DM models. Table 2 shows the different limit values of *a*(*C*_*S*_; *C*_*A*_) and *b*(*C*_*S*_; *C*_*A*_) in different possible environments for models IM and DM. For model identification purposes, we will use killing curve data obtained from batch experiments.

**Table 2:** Limit values of the switching rates in set environments for the IM and DM models. Normal and persistent population dynamics must be obtained in the different environments to calibrate the models

	*a*(*C*_*S*_; *C*_*A*_) *≈*		*b*(*C*_*S*_; *C*_*A*_) *≈*		Experiment
Environment/Model	IM	DM	IM	DM	
Exponential culture *C*_*S*_ ≫ *K* *C*_*A*_ ≪ *K’*	*a’*	*a’*	*b’ + b*_*S*_ * + b*_*A*_	*b’ + b*_*SA*_	Batch culture
Stationary culture *C*_*S*_ ≪ *K* *C*_*A*_ ≪ *K’*	*a’ + a*_*S*_	*a’ + a*_*S*_	*b’ + b*_*A*_	*b’*	
Antibiotic‐treated exponential culture *C*_*S*_ * *≫ *K* *C*_*A*_ ≫ *K’*	*a’ + a*_*A*_	*a’ + a*_*A*_	*b’ + b*_*S*_	*b’*	Killing curves obtained by treating batch culture samples
Antibiotic‐treated stationary culture *C*_*S*_ ≪ *K* *C*_*A*_ ≫ *K’*	*a’ + a*_*S*_ * + a*_*A*_	*a’ + a*_*S*_ * + a*_*A*_	*b’*	*b’*	

### Experimental set‐up

#### Bacterial strain and growth conditions

The strain used was *Klebsiella pneumoniae* CH1440, a green fluorescent protein‐tagged strain constructed after the insertion of the mini‐Tn7‐*gfp*mut3 into the genome of the biofilm‐forming CH1034 strain (Guilhen *et al*., 2015) using the method described by Choi and Schweizer (2006).

The strain was grown in M63B1 broth supplemented with glucose (4.0 g l^−1^; 1.0 g l^−1^ or 0.4 g l^−1^) and Luria–Bertani agar for CFU count. Overnight cultures were made by inoculating 15 ml of supplemented M63B1 broth in a 50 ml Erlenmeyer flask with cells from a glycerol stock (−80°C) and incubated 12 h at 37°C with shaking (100 rpm). Glucose is assumed to be the only limiting nutrient and is referred to as the substrate in the models’ equations.

#### Minimal inhibitory concentration

A solution of ciprofloxacin (Sigma‐Aldrich, Saint‐Quentin‐Fallavier, France) at 10 mg ml^−1^ was prepared. The minimal inhibitory concentration (MIC) determined according to the guidelines of the Clinical Laboratory Standard Institute (CLSI) with Mueller–Minton (MH) medium was 0.05 μg ml^−1^.

#### Killing curves

An overnight culture (12 h old) was diluted to obtain a 150 ml bacterial suspension with an optical density (OD_620_) of 0.015 in a 1 l Erlenmeyer flask. This batch culture was run for 24 h at 37°C with shaking (100 rpm).

At 0, 1.5, 3, 4, 5, 6, 7, 8, 9, 10 and 24 h, 5 ml of the batch culture was sampled and placed in a new 50 ml Erlenmeyer flask and supplemented with 1000‐fold the ciprofloxacin MIC. For each antibiotic‐treated sample, 150 μl was taken at 0 (before the addition of ciprofloxacin), 0.5, 1, 3 and 5 h. Quickly after sampling, each 150 μl sample was centrifuged at 6000 *g* for 1 min and the bacterial pellet resuspended in 1.5 ml of cold saline (water + NaCl 9 g l^−1^). Samples were then appropriately diluted with saline and plated on LB agar with an easySpiral^®^ using the exponential mode. CFUs were determined after at least 12 h of incubation at 37°C. We assumed that all bacteria able to resume growth formed a visible colony within this time.

**Figure 5:**
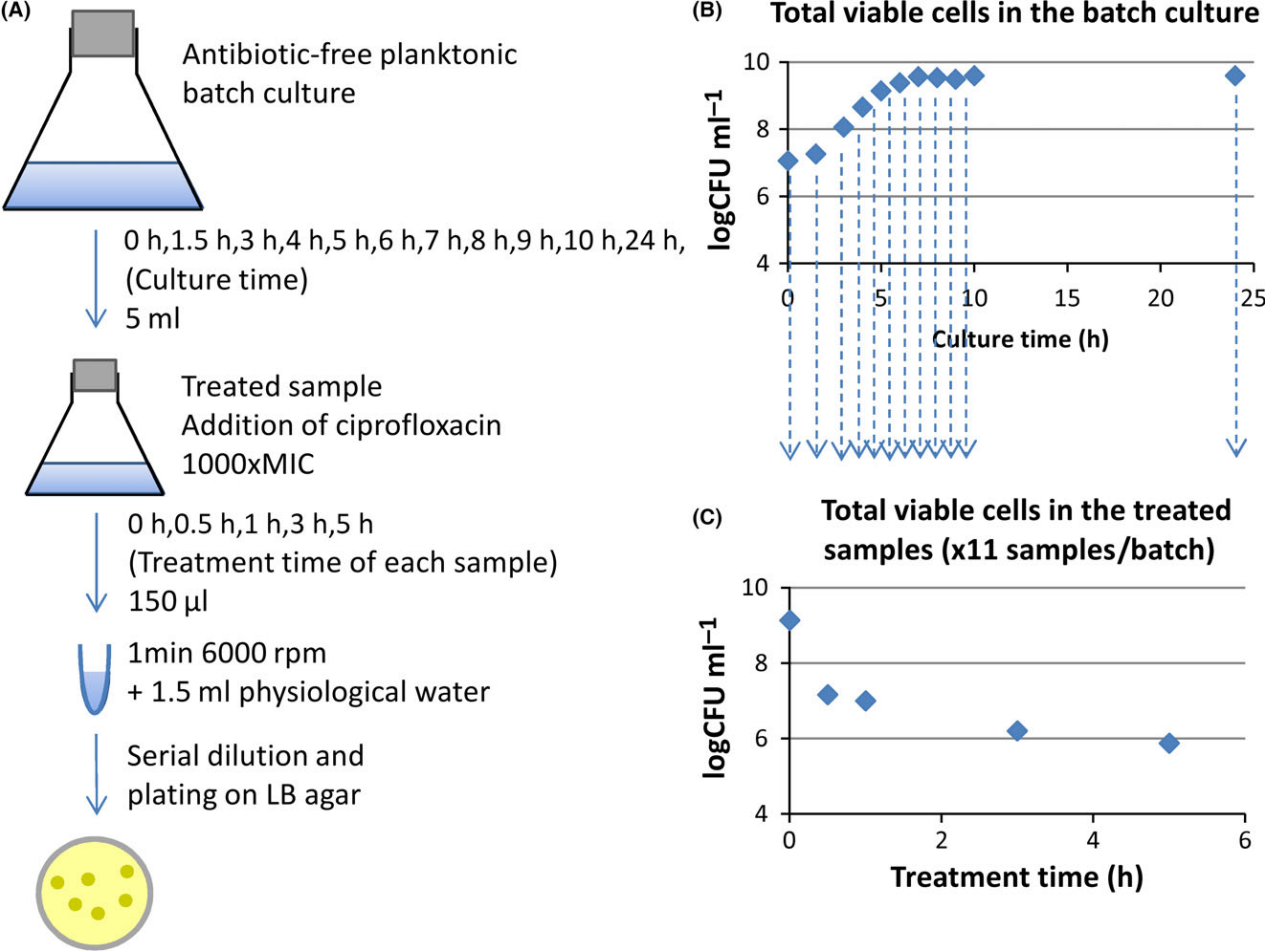
Experimental design used to obtain data to calibrate the models. A. 5 ml samples were regularly taken from an antibiotic‐free batch culture, put into a new flask and treated with 1000‐fold the ciprofloxacin MIC. Treated cultures were themselves sampled over time to determine the number of CFUs. B. The total cells in the batch cultures showed the classical phases of bacterial growth: the lag, exponential and stationary phases. C. Characteristic biphasic killing curve obtained by quantifying viable cells over time in treated samples. One killing curve is obtained for each 5 ml sample from the batch culture.

The experiment was performed three times with M63B1 broth supplemented with glucose 4.0 g l^−1^, once with 1.0 g l^−1^ and once with 0.4 g l^−1^. A schematic view of the experiments is presented in Fig. 5.

To check whether the survivors of the antibiotic treatments were resistant or susceptible to ciprofloxacin, 100 colonies were transferred on LB agar with 2.0 μg ml^−1^ of ciprofloxacin. None was able to grow overnight. A bacterial strain is considered resistant to ciprofloxacin if the MIC is > 1 μg ml^−1^ (Bonnet *et al*., 2015). The colonies transferred were from three samples plated on antibiotic‐free LB agar of three 7‐h‐old cultures treated for 5 h. Although there could be a few resistant cells, they were in minority compared with tolerant cells and we can reasonably assume that their impact on the killing curves obtained was small.

#### Quantification of the persistent population from the killing curves

**Figure 6:**
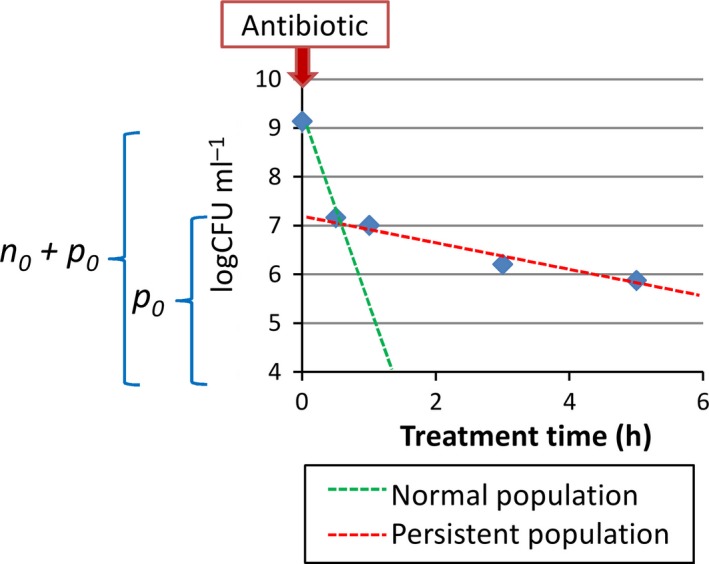
Characteristic biphasic killing curve of a population treated with a high dose of antibiotic (here 1000‐fold the ciprofloxacin MIC). The initial persister fraction in the sample, *p*_0_, can be obtained from the dynamics of the total viable cells (*n + p*) over time under antibiotic treatment.

The presence of persisters in a population leads to biphasic killing curves under antibiotic treatment (Fig. 6). During a treatment, the normal and persistent populations evolve over time as two exponentials (equations (16)).

(16) $$X\left(t\right)=n_{0}\times e^{\left(-d_{n}\times t\right)}+p_{0}\times e^{\left(-d_{p}\times t\right)}$$

*X*(*t*) is the total population over time. *n*_0_ and *p*_0_ are the initial populations of normal and persister cells in the treated sample. *d*_*n*_ and *d*_*p*_ are the decay rates of the normal and persister populations under the antibiotic treatment. The decay rates are caused by switching rates between normal and persister cells and the susceptibility of both populations to the antibiotic. The two populations are distinguished from each other because *d*_*n*_ ≫ *d*_*p*_. By fitting equation (16) to a killing curve, it is therefore possible to quantify the persister fraction in a sample. This method of quantification is used to obtain the experimental measurements of the persister fractions in the treated samples from the batch cultures.

### Optimization and validity of the models

The models were implemented in MATLAB^®^. Simulations were discretized in time and initialized with experimental data, unlike in the study of Balaban *et al*. (2004), who used analytical solutions. The optimization of the parameters was performed with the MATLAB^®^ function lsqcurvefit, which uses the least square method. The parameters were optimized to obtain the optimal fit between simulated and experimental killing curves. As the antibiotic concentrations used were very high, we approximated *C*_*A*_/(*C*_*A*_ + *K’*) ≈ 1 during simulated antibiotic treatments. We also assumed that the growth rate *μ*(*C*_*S*_) and the substrate consumed were zero during antibiotic treatments. The optimization process of the switching models is presented in the supporting information of this study (Fig. S1). Solutions obtained were the best local minima obtained from one thousand optimizations run with random initial conditions. The parameters were bounded by relatively narrow limits. The parameters *a’*,* a*_*S*_, *a*_*A*_, *b’*,* b*_*S*_, *b*_*A*_ and *b*_*SA*_ were bounded by 0 and 2 h^−1^. Parameter *K* was bounded between 0.1*Ks and 10*Ks. Parameter *k*_*n*_ was bounded by 6 and 20 and parameter *k*_*p*_ by 0 and 2 h^−1^. Experimental and simulated data were compared in log CFU ml^−1^. The experimental data used for the optimization were the means of the three experiments using the medium supplemented with 4.0 g l^−1^ of glucose. Thus, all parameters were optimized for an initial substrate concentration of 4.0 g l^−1^ in the batch culture.

We optimized the parameters of the Monod growth model separately from the switching and death parameters. We assumed that the effect of the switching models on the dynamics of the total populations was negligible. *μ*_max_ was determined by fitting the function $X\left(t\right)=y\times e^{\mu_{\max}t}$ to the exponential phase points of the batch culture, between 1.5 and 5 h (four points). *K*_*S*_, *t*_*lag*_ and *Y*_*XS*_ were then optimized by fitting the equations (13), (14), (15) to the whole growth curve.

To test the extent of the validity of the models, we run simulations by just changing the initial substrate concentration, *C*_*S,i*_, to 1.0 g l^−1^ or 0.4 g l^−1^. Simulations were initialized with the experimental normal and persistent populations at *t *=* *0 of the experiments with medium supplemented with 1.0 or 0.4 g l^−1^ of glucose. The other parameters of the models were maintained as optimized for the initial glucose concentration of 4.0 g l^−1^. The coefficients of determination (*R* ^2^) between simulated and experimental results were used to assess the validity of the models for changes in initial substrate concentrations.

## Conflict of interest

The authors declare no conflict of interest.

## Supporting information


**Fig. S1.** Optimization procedure of the switching model parameters. Simulations were discretized in time. Substrate concentration, biomass and switching rates were updated at each time step (Δ*t* = 0.001 h). *t* is the culture time of the batch and *t’* is the treatment time of the antibiotic‐treated samples. *n*(*t* = 0) and *p*(*t* = 0) are initialized with the experimental measurements. Simulations of antibiotic treatments were initialized with the variables of the simulated batch cultures at the time of the samplings. We assume *C*_*A*_/(*C*_*A*_ + *K’*) = 1 and d*C*_*s*_/d*t* = 0 during antibiotic treatments. Simulations exported sets of simulated killing curves directly comparable to the experimental ones. The parameters were optimized to obtain the best match between simulated and experimental killing curves, i.e. the smallest chi‐square possible. All parameters of the switching model selected and *k*_*n*_ and *k*_*p*_ were optimized together. 1000 optimizations with random initial conditions were tested for each model.
**Fig. S2.** Killing curves obtained with the different samples from the batch cultures with 4.0 g l^−1^ initial glucose. The error bars represent the standard deviations of three replicates. All killing curves are plotted on the same graph but were obtained from separate treated samples from the antibiotic‐free batch culture harvested at 0, 1.5, 3, 4, 5, 6, 7, 8, 9, 10 and 24 h of culture. CFUs of each treated sample were measured at 0, 0.5, 1, 3 and 5 h of treatment. The killing curve of the sample *t*0 h has a similar pattern to that of the stationary phase killing curves, with a small decay of the persistent population. There is some lag time before the persisters of the overnight cultures start to wake‐up in the fresh medium.
**Table S1.** Growth parameters optimized for the initial substrate concentration 4.0 g l^−1^.
