# Supplementary material for: Relating switching rates between normal and persister cells to substrate and antibiotic concentrations: a mathematical modelling approach supported by experiments
Source: Microb Biotechnol. 2017 Jul 21;10(6):1616–27. doi: 10.1111/1751-7915.12739 (PMC5658594; doi:10.1111/1751-7915.12739)
Supplement: Supplementary file 1 — Fig. S1. Optimization procedure of the switching model parameters. Simulations were discretized in time. Substrate concentration, biomass and switching rates were updated at each time step (Δt = 0.001 h). t is the culture time of the batch and t’ is the treatment time of the antibiotic‐treated samples. n(t = 0) and p(t = 0) are initialized with the experimental measurements. Simulations of antibiotic treatments were initialized with the variables of the simulated batch cultures at the time of the samplings. We assume C A/(C A + K’) = 1 and dC s/dt = 0 during antibiotic treatments. Simulations exported sets of simulated killing curves directly comparable to the experimental ones. The parameters were optimized to obtain the best match between simulated and experimental killing curves, i.e. the smallest chi‐square possible. All parameters of the switching model selected and k n and k p were optimized together. 1000 optimizations with random initial conditions were tested for each model. Fig. S2. Killing curves obtained with the different samples from the batch cultures with 4.0 g l−1 initial glucose. The error bars represent the standard deviations of three replicates. All killing curves are plotted on the same graph but were obtained from separate treated samples from the antibiotic‐free batch culture harvested at 0, 1.5, 3, 4, 5, 6, 7, 8, 9, 10 and 24 h of culture. CFUs of each treated sample were measured at 0, 0.5, 1, 3 and 5 h of treatment. The killing curve of the sample t0 h has a similar pattern to that of the stationary phase killing curves, with a small decay of the persistent population. There is some lag time before the persisters of the overnight cultures start to wake‐up in the fresh medium. Table S1. Growth parameters optimized for the initial substrate concentration 4.0 g l−1. [file MBT2-10-1616-s001.docx]

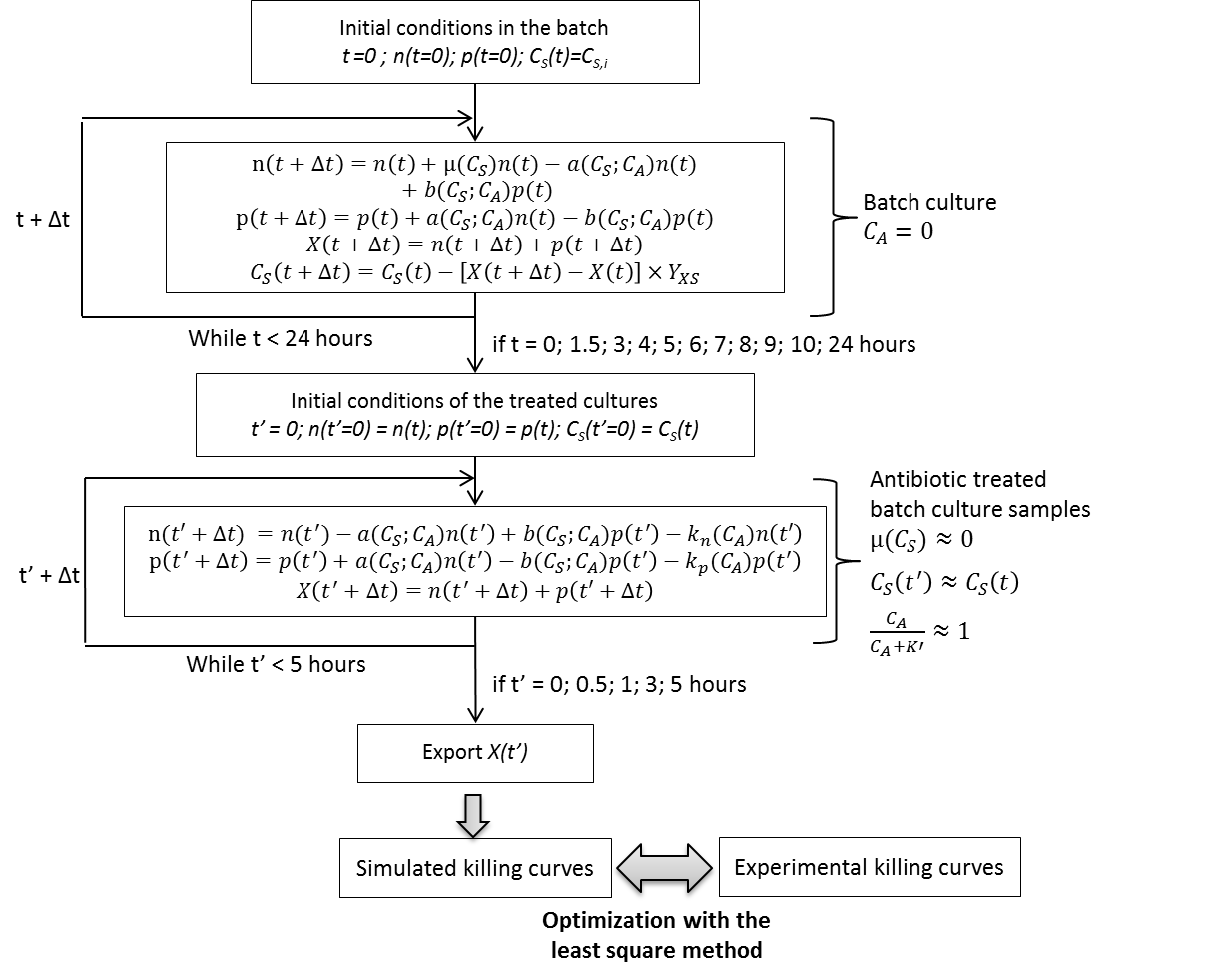


**Figure S1:** Optimization procedure of the switching model parameters. Simulations were discretized in time. Substrate concentration, biomass and switching rates were updated at each time step (*Δt* = 0.001 hour). *t* is the culture time of the batch and *t’* is the treatment time of the antibiotic-treated samples. *n(t=0)* and *p(t=0)* are initialized with the experimental measurements. Simulations of antibiotic treatments were initialized with the variables of the simulated batch cultures at the time of the samplings. We assume *C_A_/(C_A_ + K’)* = 1 and *dC_s_/dt* = 0 during antibiotic treatments. Simulations exported sets of simulated killing curves directly comparable to the experimental ones. The parameters were optimized to obtain the best match between simulated and experimental killing curves, i.e. the smallest chi² possible. All parameters of the switching model selected and *k_n_* and *k_p_* were optimized together. 1000 optimizations with random initial conditions were tested for each model.

**Figure S2:** Killing curves obtained with the different samples from the batch cultures with 4.0g/L initial glucose. The error bars represent the standard deviations of three replicates. All killing curves are plotted on the same graph but were obtained from separate treated samples from the antibiotic-free batch culture harvested at 0, 1.5, 3, 4, 5, 6, 7, 8, 9, 10 and 24 hours of culture. CFUs of each treated sample were measured at 0, 0.5, 1, 3 and 5 hours of treatment. The killing curve of the sample *t*0h has a similar pattern to that of the stationary phase killing curves, with a small decay of the persistent population. There is some lag time before the persisters of the overnight cultures start to wake up in the fresh medium.

**Table S1:** Growth parameters optimized for the initial substrate concentration 4.0g/L.

|  | **Symbol** | **Description** | **Value** | **Units** |
| --- | --- | --- | --- | --- |
| Optimized parameters | *µ_max_* | Maximal growth rate | 1.25 | h^-1^ |
|  | *t_lag_* | Duration of the lag phase | 1.13 | h |
|  | *K_s_* | Monod half-saturation constant for the substrate *S* (*µ=µ_max_*/2 for *C_S_=K_s_*). | 3.5x10^-3^ | g.L^-1^ |
|  | *Y_XS_* | Mass of substrate consumed per cell produced | 1187 | fg.cell^-1^ |
